# Improving the recognition of grips and movements of the hand using myoelectric signals

**DOI:** 10.1186/s12911-016-0308-1

**Published:** 2016-07-21

**Authors:** Gene Shuman, Zoran Durić, Daniel Barbará, Jessica Lin, Lynn H. Gerber

**Affiliations:** 1 Department of Computer Science, Volgenau School of Engineering, George Mason University, 4400 University Drive, Fairfax, 22030 VA USA; 2 Center for the Study of Chronic Illness and Disability, George Mason University, 4400 University Drive, Fairfax, 22030 VA USA

**Keywords:** Electromyograms, Machine learning, ADLs, Prehensile patterns, Classification, SAX, Dynamic time warping

## Abstract

**Background:**

People want to live independently, but too often disabilities or advanced age robs them of the ability to do the necessary activities of daily living (ADLs). Finding relationships between electromyograms measured in the arm and movements of the hand and wrist needed to perform ADLs can help address performance deficits and be exploited in designing myoelectrical control systems for prosthetics and computer interfaces.

**Methods:**

This paper reports on several machine learning techniques employed to discover the electromyogram patterns present when performing 24 typical fine motor functional activities of the hand and the rest position used to accomplish ADLs. Accelerometer data is collected from the hand as an aid in identifying the start and end of movements and to help in labeling the signal data. Techniques employed include classification of 100 ms individual signal instances, using a symbolic representation to approximate signal streams, and the use of nearest neighbor in two specific situations: creation of an affinity matrix to model learning instances and classify based on multiple adjacent signal values, and using Dynamic Time Warping (DTW) as a distance measure to classify entire activity segments.

**Results:**

Results show the patterns can be learned to an accuracy of 76.64 % for a 25 class problem when classifying 100 ms instances, 83.63 % with the affinity matrix approach with symbolic representation, and 85.22 % with Dynamic Time Warping. Classification errors are, with a few exceptions, concentrated within particular grip action groups.

**Conclusion:**

The findings reported here support the view that grips and movements of the hand can be distinguished by combining electrical and mechanical properties of the task to an accuracy of 85.22 % for a 25 class problem. Converting the signals to a symbolic representation and classifying based on larger portions of the signal stream improve classification accuracy. This is both clinically useful and opens the way for an approach to help simulate hand functional activities. With improvements it may also prove useful in real time control applications.

## Introduction

People want to live independently, but too often disabilities or advanced age robs them of the ability to perform basic activities of daily living (ADLs). ADLs are necessary personal functional activities, typically self-care, mobility, feeding, etc. They are largely performed through upper extremity (UE) movements. The hand, being the terminal UE device, is responsible for the detailed performance of ADLs and is essential for their successful completion. It is a complex part of the body that is capable of a nearly infinite number of postures and movements. Understanding the underlying physical mechanisms required for movements of the wrist and hand can help identify deficits in ADL performance with enough specificity to devise effective rehabilitation treatments that would provide many people with options for achieving and prolonging independence. That understanding can also be exploited in control applications, such as driving a prosthetic or robotic hand, and in the design of a “touch-less” computer interface.

This paper presents results of research that explores the use of machine learning pattern recognition techniques to learn and interpret the relationship between a movement and the electrical signals emitted from the muscles that control the movement. The collected signals were used to train a classifier with the aim of creating a software agent that can decide which ADL movement or movements are being performed based on a particular set of signals. The prediction accuracy of the techniques was improved by using a symbolic representation of the signal stream, incorporating a group of adjacent signals in the stream into the classification decision, and classifying entire activity segments while adopting Dynamic Time Warping (DTW) as a distance measure.

## Background

To move a voluntary muscle, the brain sends a low-level electrical, or myoelectric, signal over the central nervous system to the muscle tissue that causes contraction or relaxation, resulting in the movement. Electromyography is the study of those signals [1]. They can be measured while the muscle is contracting or relaxing and are called electromyograms, or EMGs. EMGs are very low-level — less than 10 mV (≈.0001 of U.S. household current) — and must be amplified to be measured. An electromyograph amplifies and measures EMGs and has two types of sensors: (1) needles inserted directly into muscle tissue and (2) surface sensors attached externally to the skin as close to the measured muscle as possible. Needle EMGs are inserted into the muscle and target specific areas. Surface EMGs (sEMGs), by contrast, do not distinguish between specific muscles. However, sEMG sensors have been shown to provide as good results as needle-based approaches for pattern recognition applications [2]. Their noninvasive nature and demonstrated good results make them a suitable mechanism for capturing EMG signals and are therefore the choice in this research.

The relationship between a muscle’s EMG and the resulting movement is often not obvious. Complex movements such as fine hand and finger movements usually involve several muscles working in concert, often firing sequentially, which makes finding a relationship difficult [3]. One approach to finding the relationship is to use supervised learning, or classification. In this technique a set of training instances are associated with an outcome movement to learn a classification model. The model is a function constructed from the training instances that approximates some true underlying relationship. When presented with a similar, but previously unseen instance in the future, the model (the learned function) is used to predict the outcome by translating the set of new signals into the appropriate grip or movement.

## Related work

Relating EMGs to movement has two basic use cases. One involves control applications in which the EMG readings are used to drive a prosthesis, robotic hand, or a touchless computer interface. These tend to be real time applications that require that the signals be acquired and processed, and a related activity initiated in a very short time frame, often a fraction of a second. The second involves using EMGs to assess motor and sensory signals. Abnormalities of the signal may contribute criteria that may assist in making diagnoses or tracking recovery. Additionally, research has provided data about the relationships between signal amplitude and muscle strength. The relationships are complex, but may provide some clinically relevant information [4]. This second use case is usually done as a batch process that allows for the complete collection of a set of EMG data that can be read and interpreted at a later time.

Much of the related work involves the first use case: exploiting EMG patterns in myoelectric control system (MES) applications [5], especially those needed to drive a prosthetic hand or arm. Early efforts involved using classification to recognize EMG patterns for as many as six gross movements of the arm or wrist [6–8]. Others concentrated on recognizing movements of the hands and fingers for up to 12 movements using up to 32 sensor channels [9, 10]. Still others combined the recognition with also predicting force [11, 12]. Accuracies ranged from 50 % up to the high 90s.

More recently the Ninapro project [13] used classification to recognize up to 52 grips and finger postures with the aim of driving a prosthetic hand. That effort employed 12 sensors, eight placed uniformly just below the elbow, the remainder on the extensors, flexors, and biceps. Overall accuracy of the classifier was in the 50–75 % range. The collected data are publicly available.

The research reported in this paper differs from previous efforts in that it attempts to recognize a set of fine motor movements of the hand needed to perform typical ADLs. It uses a moderate number of sensors targeted to specific muscle areas. It reports on results from classification using several well-known classification techniques. A symbolic representation scheme for the sensor data is employed and the concept of an Affinity Matrix is introduced to construct a learning model using adjacent signals in the stream and perform classification. Finally, Dynamic Time Warping (DTW) is used as a distance measure in a nearest-neighbor classification scheme.

## Methods

sEMGs were recorded while a subject performed upper extremity (UE) movements used in a selected set of ADLs. The grips and movements selected for this research focused on those of the hand and wrist executed in a short time span (five seconds or less). They involve fine motor movements required to perform typical activities of daily living and include several types of grips and associated movements: lateral (key) grip (gripping and turning a key), power or hammer grip, door knob grip and turn, jar lid grip and turn, scissors grip and open/close, 3-jaw chuck grip and tip pinch grip [14].

The 25 activities selected for study in this research include the 24 specific grips and movements and the neutral/rest position listed in Table 2. The Table includes their description, codes (used to label the activity) and action group or grip family. The eight action groups are determined by the base hand grip that must be engaged before the related follow-on actions are performed. Goals include exploring the ability to recognize the 25 activities from their EMG signal patterns, determining relationships between grip signals and their follow-on movements, and discovering relationships among the various action groups’ EMG signals.

### Instrumentation

The DelSys Trigno Wireless™ sensors and base station were used for sEMG and accelerometer (ACC) signal collection [15]. The sensors each contain a rechargeable battery that communicates with the base station at a range of up to 40 m. The base unit communicates with the DelSys EMGWorks™ Acquisition package via a USB interface that, in turn, drives the collection and control of the sensor signals and allows for the real-time monitoring of the signal. sEMG signals were collected at rate the of 2 kHz, ACC signals at 148.1 hz.

### Sensor placement

Ten sensors were used in the data collection, each attached to the skin surface of the subject’s hand and arm used in performing the actions. The sensors were secured using adhesive skin interfaces provided by DelSys for the purpose. Eight were sEMG sensors located over the arm muscles believed to contribute to the grip or movement. Seven were located on the extrinsic muscles in the forearm that control the hand and wrist, and one on the biceps. A sensor was placed on the biceps in an attempt to capture the contributions of the upper arm to the activities in Table 2, especially those involving raising an object and turns of the hand and wrist. Trials conducted during previous research [16] indicated measurable EMGs from the biceps but not the triceps during the listed activities. Guidance from [1] indicated good candidate areas of the arm contributing to hand and wrist movements. One (on the extensor indicis) was configured to also collect ACC data for possible later use. EMG data from the eight sensors are used as classification features.

Two additional sensors were attached on the active hand’s posterior, just below the base of the thumb and below the little finger. They were configured to collect ACC data as an aid in labelling the movement actions later. Changes in the ACC data from the hand sensors indicate that a movement has started and the associated EMG data instances can be appropriately labelled. The location on both sides of the hand was chosen to maximize the detection, while locating them on the hand’s posterior minimized interference with the subjects’ performance of the actions. Data from these two sensors are not used as classification features.

**Fig. 1:**
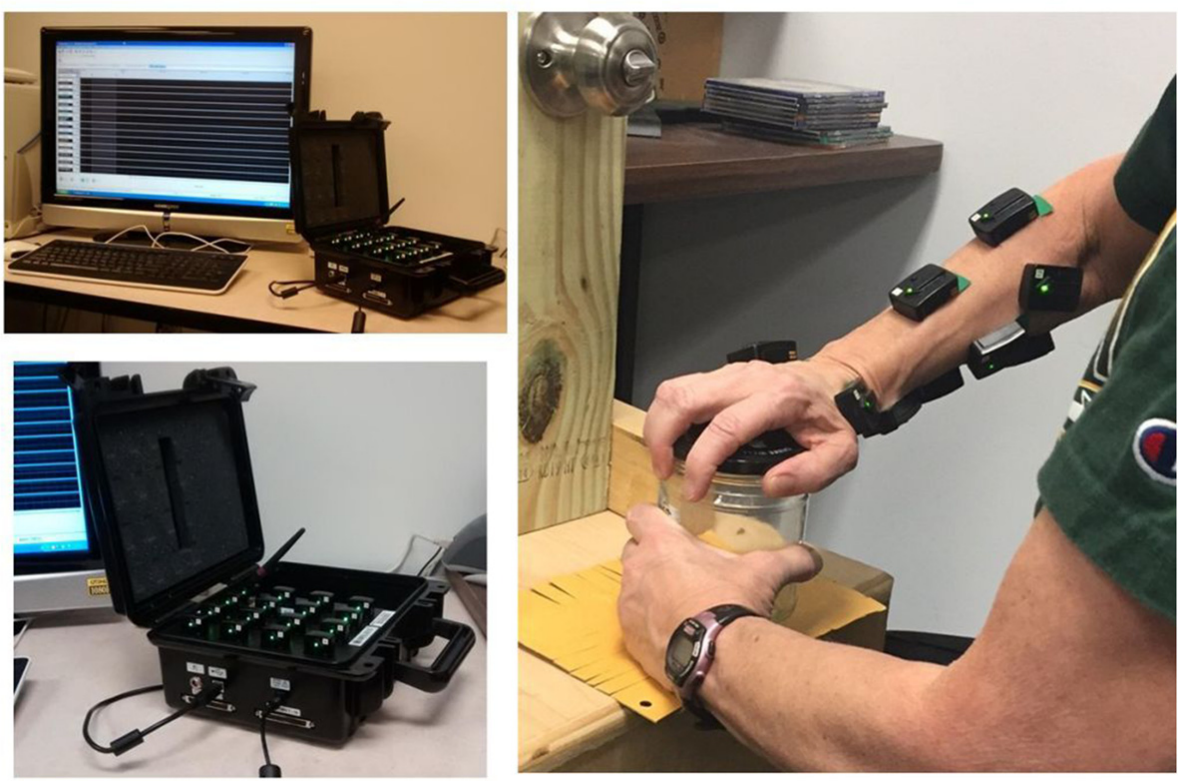
Trigno kit used for data collection (*left*, *top* and *bottom*) and a subject (*right*) performing a jar lid turn with ten sensors attached to the action arm

Sensor placement is shown in Table 1 and was not altered during the trials. Figure 1 shows the Trigno kit and a subject turning a jar lid with ten sensors attached to the action arm.

**Table 1:** Location of the ten sensors and data collected

Sensor#	Muscle location	Data collected
1	extensor digitorum (ED)	EMG
2	extensor indicis (EI)	EMG and ACC
3	flexor carpi radialis (FCR)	EMG
4	flexor digitorum superficialis (FDS)	EMG
5	flexor carpi ulnaris (FCU)	EMG
6	pronator quadratus (PQ)	EMG
7	brachioradialis (Bra)	EMG
8	biceps brachii (Bic)	EMG
9	base of the thumb (posterior)	ACC
10	base of little finger (posterior)	ACC

**Table 2:** The activities (grips and associated movements) performed as part of the data capture protocol

Grp#	Action group	Code	Description
1	hammer	HG	hammer grip
1	hammer	HR	hammer raise
1	hammer	HGR	hammer grip - raised pos.
1	hammer	HL	hammer lower
2	tip pinch	TPG	tip pinch grip
2	tip pinch	TPR	tip pinch raise
2	tip pinch	TPL	tip pinch lower
3	3-jaw chuck	3JCG	3-jaw chuck grip
3	3-jaw chuck	3JCR	3-jaw chuck raise
3	3-jaw chuck	3JCL	3-jaw chuck lower
4	key	KG	key grip
4	key	KTS	key turn - supination
4	key	KTP	key turn - pronation
5	scissors	SCG	scissors grip
5	scissors	SCO	scissors open
5	scissors	SCC	scissors close
6	door knob	DKG	door knob grip
6	door knob	DKS	door knob turn - supination
6	door knob	DKP	door knob turn - pronation
7	jar lid	JLG	jar lid grip
7	jar lid	JLS	jar lid turn - supination
7	jar lid	JLP	jar lid turn - pronation
8	ball	BG	ball grip
8	ball	BSQ	ball squeeze
-	All	NR	neutral/rest

The activity code is used as the class label and the 8 action groups are the base hand grip that’s engaged before the related follow-on actions are performed

### Data collection protocol and feature creation

Data were collected from three subjects as described below. The subjects included one middle aged male, one middle aged female, and one male in his 20s. All were in good physical condition, without disability of any kind, and able to perform all grips and actions without difficulty. All were naturally right handed and used their right hand to perform the activities. The collection followed a protocol approved by the George Mason University Institutional Review Board, Reference number 8672.

The subjects performed a series of eight two minute data collection runs. The activities for a single action group listed in Table 2 were performed in one run. Note that there are eight action groups, one group per run. The subjects followed a timed script in performing 12 repetitions of the action group’s grip and movements, each repetition lasting ten seconds.

Each two minute run starts with the subject maintaining their hand and arm in a neutral or rest (NR) posture for the first five seconds. At second five the repetitions begin. First, the grip for the action group was engaged and held for two to three seconds. At the eight second mark, the second action in the group is performed for one second. What happens during the next second depends on the group. For some, a third action is performed, for others the second action is sustained, and for the rest the neutral/rest posture is maintained for one second. At second ten the last action in the group is performed for one second, followed by four seconds of neutral rest. This is repeated 12 times for each two minute run.

The subjects were instructed to begin each grip with the hand in proximity to, but not touching the object to be grasped, ensuring the act of gripping was captured. For the hammer, ball, scissors, and jar lid action groups, the object was placed in the non-active hand between repetitions, with the active hand approximately ten centimeters from the object. For the two action groups involving turning, the door knob and key, the subject was instructed to release the object after the first turn movement and resume the grip before proceeding with the second turn, essentially inserting a brief neutral/rest between the two turns. For the fine movement groups, tip pinch and 3-jaw chuck, the subject was instructed to grasp the objects, a U.S. quarter dollar coin for tip pinch and golf ball for the chuck, with enough force so that it would not be dropped if the hand were lightly slapped. Apart for the above the subjects were allowed to choose the way in which they performed the actions.

Table 3 illustrates the protocol followed for all eight action groups. Each group is performed as one set of 12 repetitions over a two minute time span. A single repetition lasts 10 s and starts on second 5, 15, 25,... 115, within each 120 s interval. For the hammer group, for example, the hammer grip (HG) is performed for two or three seconds (*s*_*x*_5 - *s*_*x*_6/*s*_*x*_7), followed by a hammer raise (HR) for one second, hammer grip in raised position (HGR) for one second, then hammer lower (HL) for one. The activity for second seven, *s*_*x*_7, varied between the indicated activity (e.g., HG) and neutral/raise (NR) since the grips are not distinguished by accelerometer data and their termination could therefore not be precisely fixed. Likewise, the activities occurring in second nine (*s*_*x*_9) for four of the groups were a hybrid of the activity and NR. The remaining time until the start of the next repetition is the neutral/rest (NR) position for approximately 4 s (5 for the ball group). The columns *s*_(*x*−1)_4 and *s*_(*x*+1)_15 are included to show the action immediately before and after a repetition.

**Table 3:** Data collection activities

*One 10 s repetition*									
Act. group	*s*_*x* − 1_4	*s*_*x*_5	*s*_*x*_6	*s*_*x*_7	*s*_*x*_8	*s*_*x*_9	*s*_*x*_10	*s*_*x*_11 − 14	*s*_*x*+1_15
hammer	NR	HG	HG	HG/NR	HR	HGR	HL	NR	HG
tip pinch	NR	TPG	TPG	TPG/NR	TPR	TPR/NR	TPL	NR	TPG
3-jaw chuck	NR	3JCG	3JCG	3JCG/NR	3JCR	3JCR/NR	3JCL	NR	3JCG
key	NR	KG	KG	KG/NR	KTS	NR	KTP	NR	KG
scissors	NR	SCG	SCG	SCG/NR	SCO	SCO/NR	SCC	NR	SCG
door knob	NR	DKG	DKG	DKG/NR	DKS	NR	DKP	NR	DKG
jar lid	NR	JLG	JLG	JLG/NR	JLP	JLP/NR	JLS	NR	JLG
ball	NR	BG	BG	BG/NR	BSQ	BSQ	NR	NR	BG

The table shows one action group ten second repetition (columns *s*_*x*_5 through *s*_*x*_14, one second per column) for each of the eight groups in the protocol, covering the performance of all 25 activities. *s*_*x*−1_4 and *s*_*x*+1_15 are shown to illustrate the second immediately before and after each repetition. The text gives a detailed explanation

An attempt was made during data collection to ensure the synchronization of the actions with the indicated times and durations. The EMGWorks™ timer was used to prompt the subject for the next action. However, this could only be done to a certain level of precision and so the timings, while within a few hundred milliseconds of the stated values, should be regarded as approximate.

Each subject’s data collection resulted in eight separate files, one for each action group. Each file contained the signal data (EMG and ACC) for one two minute run including 12 repetitions for the group. The DelSys EMGAnalysis™ package was used to visualize and process the collected signal sequences. The Trigno sensors filtered the signals during collection with a 20–450 hz bandwidth using a flat Butterworth filter to preserve EMG signal amplitude and phase linearity. This eliminates noise while capturing most of the signal [17]. The mean absolute value (MAV) was computed for each sequence, specifying a collection window of a 100 millisecond signal segment with a 50 ms overlap. The window size was selected to allow for quick classification decisions needed for real time control applications. The trade-off of varying window sizes versus accuracy is discussed in [5] and [8].

To compute the MAV for the specified window size, let *f*_*j*_(*iT*_1_),*i*=1,2,… be the sampled data for channel *j*=1,…,8; *T*_1_=1/2000 second is the sampling period. *g*_*j*_(*kT*),*k*=1,2,… is sampled filtered data computed using Mean Absolute Values (MAVs) for windows of width 2*T* at steps of size *T* using

1 $$ g_{j}(kT) = \frac{1}{2N} \sum_{i=-N+1}^{N} \left|f_{j}(kT+iT_{1})\right|, \;\;   $$

where *T*=1/20 second, *N*=100, *j* is the sensor channel index, and *k* the sample index.

**Fig. 2:**
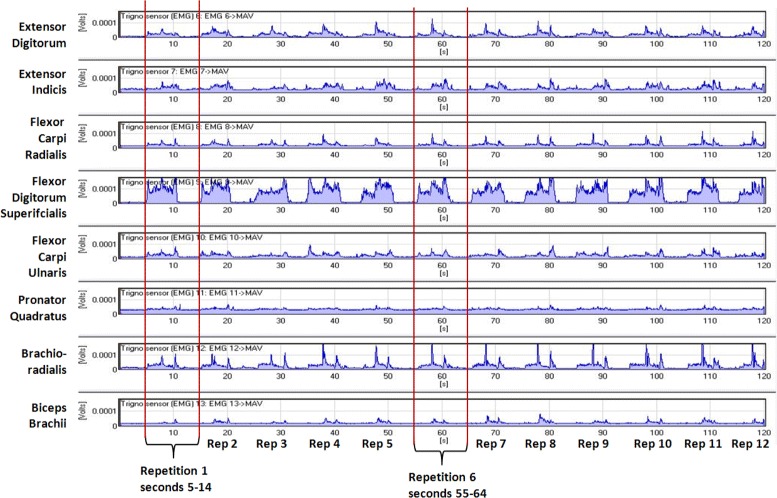
Graph of the mean absolute values (100 ms. window) for the eight sensor channels for an entire 120 s hammer group data capture run for one subject. A vertical slice from the repetition noted along the x-axis (the slice for the 1st and 6th are shown) up through the eight sensor graphs reveals the pattern for each repetition. Each repetition starts at seconds 5, 15, 25, …with a grip (HG), followed by raise (HR), grip in raised position (HGR), *lower* (HL), and a short rest (NR) before starting the starting next repetition

**Fig. 3:**
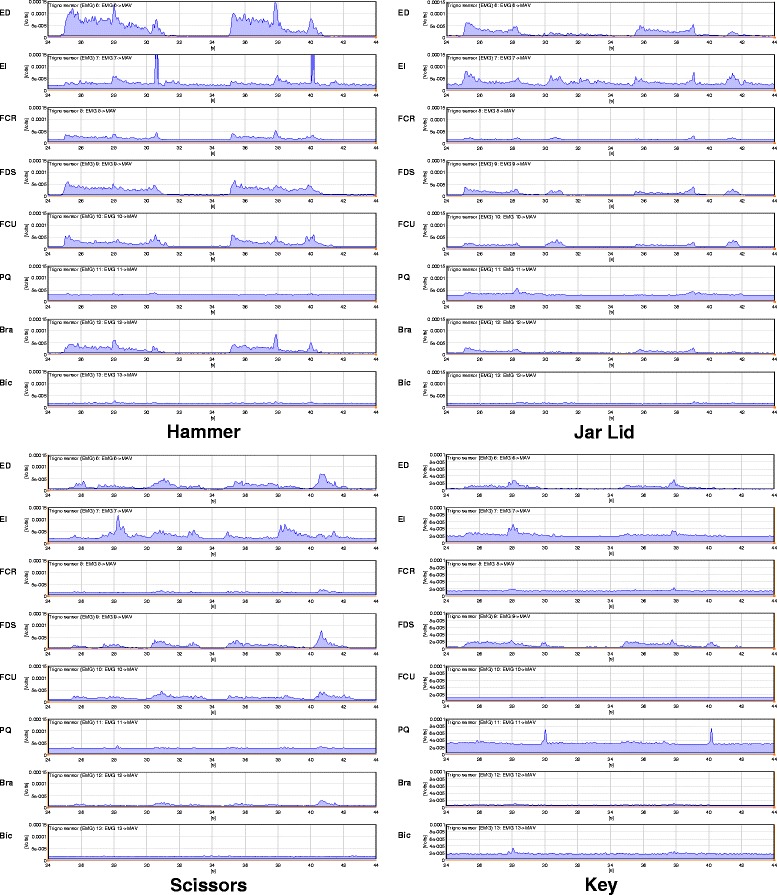
Graph of four of the eight action groups mean absolute values - 20 s interval (24–44) covering two complete repetitions (3rd and 4th) of each group’s grip and movements. The four represent different types of grips: power (hammer), precision (jar lid), dynamic tripod (scissors), and precision handling - small objects (key). Pattern differences can be observed among the four. Note that the Key movement requires less force than the other three and shows a lower signal value despite its graph being scaled at a *lower* value than the others. Abbreviations for the muscle signal channels are shown on the *left* of each graph row

An 8-tuple *g*_*j*_(*kT*),*j*=1,…,8 computed using Eq. (1) is one training instance. The result was 20 training instances per second, or 2400 EMG instances per 120 s data collection run. The Trigno captures the ACC data at a different frequency and the calculation results in 21.1 instances per second, or an additional 132 for the 120 s run. EMGAnalysis™ macros were used for this processing. Figure 2 shows the MAV of eight sensor signals for the one entire 120 s stream for one hammer group data capture run. Figure 3 shows a 20 s, two repetition sample for four of the action groups.

Data for all eight files for each of the subjects’ collection runs were processed and labelled. Labels were assigned by reviewing the signal stream at the beginning of each of the action repetitions, seconds 5, 15, 25,... 115. The stream consists of the elapsed captured time as well as the MAV of the signal values. The grips were assumed to start on the indicated times and were labelled accordingly. The start of subsequent movements within each repetition were indicated by a change in ACC signals for the two sensors on the hand. Since the ACC data were collected at a different frequency, the elapsed times do not exactly match the EMG times on a one-to-one basis. The ACC values were matched up with the closest EMG value based on the elapsed time of each, the difference never being more than 30 milliseconds. The summed difference of the ACC values between time *t* and *t*+1 were computed. The sum exceeding double the median value for the entire run was interpreted as being the start of an action and the EMG 8-tuple closest to the time instance was assigned the appropriate movement label.

Establishing the onset of the grips was a little more difficult since they are static activities and are not as clearly distinguished by a change in ACC readings. However, since the subjects were required to begin each grip with their hand off the object and move it to the object to establish the grip, there was a more modest ACC change as the grip was assumed. The end of a sequence of ACC changes during an elapsed time in which a grip was expected was used to label a data instance as the start of a grip and sustain it for up to three seconds. The result was that some label instances varied by a few processing windows before or after the expected time synchronization points indicated in Table 3. While this was never more than a 500 ms adjustment before or after the five second mark, the inexactness of the labelling process may have led to some misclassification at the onset of a static grip activity. While the process described above resulted in superior labelling compared with that used in [16], it remains a difficulty, has been reported elsewhere in the literature, for example [13], and as yet does not have a good solution.

As can be seen in Table 3, the NR (neutral/rest) samples greatly outnumber the others. In order to maintain an approximate balance among the 25 classes, the number of NR samples was reduced by including only the samples between seconds one and three of each run. This resulted in ≈60 (3×20) NR samples per run instead of ≈960, and an overall reduction of ≈7,680 to 480 for all eight runs for a single subject. The NR instances outside the range of zero to five seconds occupy a gap in data collection between activities or activity repetitions during which the subject was only sometimes in the NR posture. During those gaps subjects occasionally performed some movement to relax or get ready for the next repetition. Because of this uncertainty, those NR instances could not be uniformly labelled correctly and are ignored. By contrast, subjects always started a run in the neutral/rest position and so sampling at seconds one to four ensures those instances are truly NR and do not include casual or unintended movements.

Each of the labelled actions was truncated to a maximum of 40 100 ms segments in length. This was done to facilitate the use of a technique called Dynamic Time Warping as a distance measure in one of the learning approaches, which will be explained later. To ensure uniformity in evaluating the learning techniques, all approaches described in the next section use the dataset as processed in this way.

### Learning approaches

Three approaches were used in learning the sEMG patterns leading to the recognition of selected grips and movements. The first uses several well-known classification techniques operating on 100 ms MAV windows as learning instances. The second two use a symbolic representation of the signal stream that divides it into discrete ranges. One of the two creates an affinity matrix to model the learning instances and employs nearest neighbor classification. The approach takes advantage of the time-series nature of the data stream by using a selected number of instances immediately before the one being classified to help make the classification decision. The other uses Dynamic Time Warping [18] as a distance measure in conjunction with nearest neighbor. Instead of treating each 100 ms instance in isolation, this approach considers all the instances that constitute an entire labelled activity to make classification decisions.

**Classification (approach 1)** The key point in classification is the use of a class label for each training instance that must be manually assigned [19]. Here, a label is assigned to each 100 ms MAV window of each grip or movement, including the neutral/rest posture. A labelled 100 ms window is one training instance. Table 2 shows the code labels used to track the grips and movements. The label allows the classifier to build relationships between signals and the class (the grips and movements). The 25 grips and movements form a 25-class problem in which a classifier is trained to recognize all 25 from their sEMG signal patterns. The classifiers were trained and tested on their ability to recognize the class of each 100 ms instance in isolation, without considering any time sequence dependencies among them.

Classifiers are measured on the accuracy of their predictions. Accuracy is the percentage of test instances correctly identified from the total number evaluated. For multi-class problems, how well the classifier recognizes instances of each class - measured by the true positive rate (TPR) or Recall - is also of interest since it can vary.

After labelling, the data were normalized before applying the approaches described below. Each of the eight channels was individually normalized by subtracting the channel value mean and dividing by its standard deviation. The eight means and standard deviations of the training data channels were used to normalize held out test data.

Several classifiers were tried, including Decision Tree, Random Forest (RF), Support Vector Machine (SVM), and Nearest Neighbor (NN). The Weka toolset, v 3.6.11 [20] was used to perform all classification in this first approach. The Weka default values were used except as follows.

The Decision Tree used the Weka J48 implementation of the C 4.5 decision tree algorithm. RF is an ensemble classifier that generates a stated number of trees using a subset of randomly chosen features for each generated tree. A voting process in which each of the generated tree’s choice is tallied determines the winning grip or movement. The number of RF trees generated was varied from 15 to 100, with performance levelling off at 25. The RF results reported in this paper were therefore generated using 25 trees. For *K*-nearest neighbor (*K*-NN), the normalized Euclidean distance measure was used, and the value of *K*, the number of neighbors used to determine the class, was varied from 1…5. 1-NN performed best and is reported.

While the aforementioned classification methods handle multi-class problems as part of their core algorithms, SVM is inherently binary. The Weka implementation employed here uses a 1-versus-1 approach and an implementation of the Hastie and Tribshirani pairwise coupling method [21]. For *C* classes, *C*(*C*−1)/2 classifiers are built. The pairwise class probability estimates are combined into a joint estimate for all classes and used to predict the class.

The SVM parameters were evaluated and set using a grid search. The Polynomial, RBF, and Pearson Universal Kernel (PUK) functions were tested. For the Polynomial kernel, the exponent parameter was tested at 2 and 3, with no improvement over the default of 1. The RBF kernel bandwidth parameter, *γ*, was tested for 0.01,0.05, and 1.0. The PUK parameters were tested for *ω* and *σ* values of 0.25,0.5,1, and 5. The SVM regularization parameter, *C*, was tested for values 0.5,1,5,10,25,50, and 100, with accuracy leveling off at 5. The PUK kernel with *ω*=*σ*=0.5 and *C*=5 were found to result in the highest accuracy when tested using data for the three subjects. These are the SVM settings used to produce the results reported in this paper.

Stratified ten-fold cross validation was performed using all training data to select parameters and evaluate classifier performance. Stratification ensures that a representative proportion of instances of each class is included in each of the ten folds.

**SAX** SAX (Symbolic Aggregate approXimation) is a method of representing a time series using a set of symbols assigned based on a discrete range of the sensor values [22]. It was used in the second and third learning approaches and is described in this section. The remaining two learning approaches are described in the following two sections.

Since the signal stream coming from the performance of a grip or movement represents a time series, trying the SAX approach to represent the signals was appropriate. Only the idea of symbolic representation of the signal rather than real number values was borrowed from SAX, an approach that includes other concepts not used here. Since there are eight signal sensor values, each one of the eight 100 ms MAV values is converted to an 8-tuple symbol for use as a feature. As with the MAV values, the result is 20 SAX 8-tuples per second. While the feature dimension remains at eight, the total feature space of possible values is reduced to a finite number determined by the size of the alphabet of symbols.

**Fig. 4:**
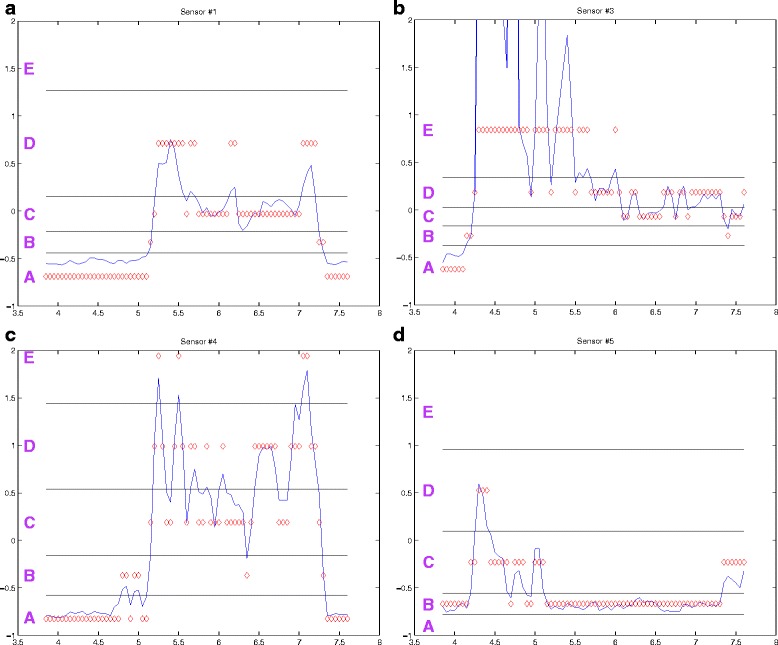
Assigning symbols to the five sensor streams using an alphabet size of five. Graphs for four of the eight signal channels are shown for a 3.5 to 8 s segment, corresponding to the end of the neutral/rest (NR) activity and the entire hammer grip (HG), for the average of all sensor values for the training runs. The SAX window size is 100 ms. The horizontal lines show the cut-off boundaries for the five symbol alphabet, A through E. The selected graphs show the diversity of cut-off values that vary for each of the eight sensors

In this implementation of *n*-symbol SAX the range of possible signal values is divided into *n* intervals in such a manner that all symbols are equally probable. For each channel the signal probability is estimated using the histograms of signal values. An example of applying this method to discretize signal data is shown in Fig. 4. Each SAX window covers 100 ms, one MAV segment per window. In the remaining two learning approaches the alphabet size *n* was varied between 5 and 15. Since each 8-tuple signal value is replaced by an 8-tuple *n*-value symbol, a total of *n*^8^ different 8-tuples are possible. MATLAB^®;^ code was developed to implement the above as well as the second and third learning approaches described in the following two sections.

For an *n*=5 size alphabet, examples of SAX 8-tuples are ‘AAAAAAAA’, ‘ABDEDBAC’, ‘CDEEDCDE’, and ‘ABCDEEDC’. In the remainder of this paper a SAX 8-tuple of symbols is understood to represent one 100 ms MAV signal segment and will be referred to as a word.

**Fig. 5:**
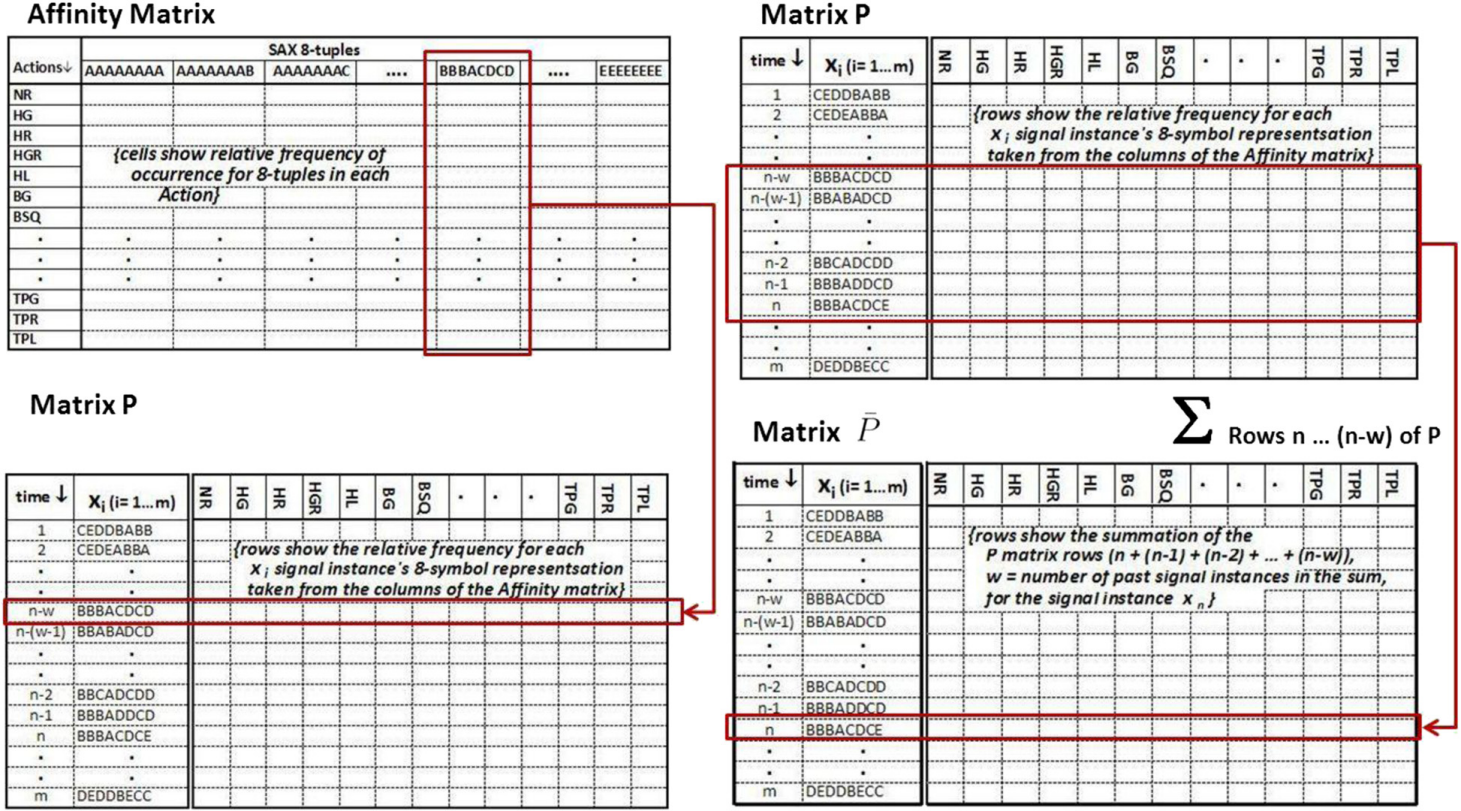
*Left* shows creation of the *P* matrix from the Affinity matrix. *Right* shows the creation of $\bar {P}$ from the *P* matrix with an affinity summation window of *w* (summation of the current classified instance plus the previous *w* rows) as described by Eq. 3

**Affinity matrix (approach 2)** One of the key ideas in this approach is the concept of building a class affinity matrix *A* from the training data and using it as the classification model. The matrix has one row for each of the 25 class actions listed in Table 2. The 100 ms MAV 8-tuples in the training dataset are converted to SAX words as described in the previous section. The words are used to create the matrix columns, each column consisting of a unique word encountered in the training dataset. The cells are the relative frequency, or affinity values, in the training data that each word is associated with the corresponding class rows. As a stream of words to be classified is processed, the column for that word is looked up in the matrix and, if found, recorded in a new matrix, *P*. If not found, this new word is written into the matrix and its matrix column and row in *P* populated with the column values for the closest word already in the matrix. Finally, as each new word is encountered and entered in *P*, the class activity decision is determined by summing the value of the affinities for the current occurrence and the affinity values for the previous *w* occurrences, where *w* is a parameter that was varied in several trials. The values are recorded in a separate matrix, $\bar {P}$, which holds the stream of summed affinity values. The class action decision is the maximum affinity value in the $\bar {P}$ row for the current occurrence. This is described in more detail below, and illustrated in Fig. 5.

To build *A*, from the training dataset *A*_*ac*_ is computed as the number of times a word *c* occurs in action/class *a*. $\bar {A}$ is computed by normalizing *A* such that each row sums to 1, i.e.

$$\bar{A}_{ac} = A_{ac}/\sum_{c=1}^{N}A_{ac}, \forall a,c $$

From $\bar {A}$, $\hat {A}$ is computed by normalizing columns of $\bar {A}$, i.e. by making columns of $\hat {A}$ into unit vectors. *N* is the number of distinct words found in the training dataset. Note that *N* is usually less (much less for *n*>4) than the total space of all possible words.

$\hat {A}$ is used in the recognition phase. Let the signal samples to be classified be **x**_*i*_,*i*=1,2,…*m*, where *m* is the number of instances, converted to words, presented for classification.

A matrix *P* is created one row at a time, each row corresponding to a newly encountered word. The *i*-th row *P*_*i*_ of *P* is the *c*_*i*_th column $\hat {A}_{c_{i}}$ of $\hat {A}$, where *c*_*i*_=**x**_*i*_ or the nearest word to **x**_*i*_. The classification step simply estimates the class *a*_*i*_ as

2 $$ a_{i} = \text{argmax}\{P_{i}\},   $$

i.e. finds the class corresponding to the index of the largest value in the row *P*_*i*_.

These values can be quite noisy since they treat all word instances in isolation. An improvement involves taking into account the time series dependency of adjacent signal instances. Instead of using *P* for recognition, create $\bar {P}$ by summing the affinity values in the rows of *P* occurring immediately before the particular instance of *P* currently being classified. The number of values to be summed is determined by a window parameter *w*. For an instance *x*_*i*_ to be classified that’s part of a sequence containing *m* instances, the predicted class for the *i*th instance in the sequence is computed using the *w* affinity values of the rows between *i*−*w* and *i* as follows

3 $$ \bar{P}_{i} = \left\{ \begin{array}{ll} \sum\limits_{j=1}^{i}P_{j}, &0 < i \le w \\ \sum\limits_{j=i-w}^{i}P_{j}, &w < i \le {m}\\ \end{array} \right.   $$

Note that in Eq. (3), the first (*w*−1) values in the sequence have less than *w* values and must be handled as a special subcase.

The predicted class *a*_*i*_ is estimated from $\bar {P}_{i}$ as in Eq. (2).

$\bar {A}$ and $\hat {A}$ were estimated by computing affinity values of unique words and activity classes. The columns of $\bar {A}$ and $\hat {A}$ correspond to unique words in the training set, and each column *j* corresponds to a unique word *s*_*j*_. Call this set *S*. Given a signal 8-tuple **x**_*i*_ to be classified, if its corresponding word *s*_*i*_ exists in $\hat {A}$ it is added as a row to *P*_*i*_ and $\bar {P}_{i}$, and to recognize the class *a*_*i*_ using Eqs. (2-3). If *s*_*i*_ does not appear in $\bar {A}$, the set $\phantom {\dot {i}\!}S_{i}=\{s_{i_{1}},s_{i_{2}},\ldots \}$ of symbols is found in *S* which are closest to *s*_*i*_ using lexical distance $d_{l}(s_{i},s) = \sum _{k=1}^{8}|s_{i}(k)-s(k)|$ for symbols *s*_*i*_ and *s*. Given *S*_*i*_, columns $\phantom {\dot {i}\!}\bar {A}_{i_{1}},\bar {A}_{i_{2}},\ldots $ are added corresponding to symbols *S*_*i*_ to form a vector **p**_*i*_. **p**_*i*_ is normalized to 1 and transposed to form the row *P*_*i*_ which is then used for recognition using Eqs. (2–3).

Figure 5 illustrates the structure of the Affinity and *P* matrices as well as their relationship. Matrix $\bar {P}$ has the same structure as *P* except that the values of $\bar {P}$ are created using the affinity summation scheme as described by Eq. (3).

The effect of the affinity summation scheme is to change the prediction of class “X” found in the middle of a long sequence of class “Y” by taking advantage of the temporal context information inherent in time sequences. In many instances, noise in the data can introduce clearly wrong predictions that this process corrects. Here the size of the summation window was varied in an attempt to find an optimum balance between an accurate prediction of a grip or movement sequence and limiting the size of the window. Larger values of *w* result in higher accuracies, but require that more information be known about the sequence. Since this technique uses information that occurs before the instance to be classified, it is suitable for use in real-time applications since no "future" information need to be seen prior making a classification decision.

**Dynamic time warping (approach 3)** The Affinity Matrix approach attempts to take advantage of the time series nature of the signal data by considering the context in which a 100 ms SAX 8-tuple instance (word) occurs. Specifically, it takes into account the prediction of the current instance under review, *x*_*i*_, and the previous ‘n’ instances. The approach described in this section also attempts to exploit the sequential nature of the data. Here, however, the shape of the entire activity (e.g., hammer raise, ball squeeze), which consists of a sequence of words, is matched against the patterns in the training data to find the corresponding action by using Dynamic Time Warping (DTW) [18, 23, 24] as a distance measure. A short description of DTW is given below, followed by the specifics of how it is used in this third learning approach.

**Fig. 6:**
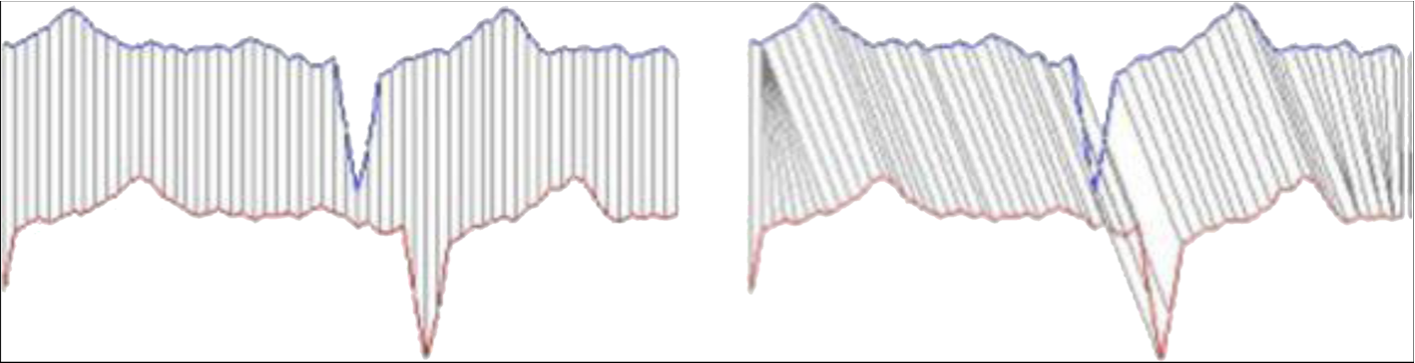
(*Left*) Alignment for Euclidean distance between two time series. (*Right*) Alignment for Dynamic Time Warping (DTW) distance between two time series. For DTW a predetermined warping window parameter is set to govern the width of the path for comparison between the two series. Adapted from [24]

Euclidean distance is a well-known distance measure in which sequences are aligned in a point-to-point fashion, i.e. the ith point in sequence Q is matched with the ith point in sequence C. Its simplicity and efficiency makes it a commonly used distance measure. While it often works well, it requires that both input sequences be of the same length and is sensitive to shifting along the time axis. For example, the top and bottom time series in Fig. 6 appear to be very similar. In fact, the time series below is the shifted version of the time series above. However, the slight shifts along the time axis will result in a large distance between the two time series.

Such a problem can generally be handled by more flexible distance measures such as DTW. DTW uses dynamic programming to determine the best alignment to calculate the optimal distance. The warping window width parameter determines how much warping is allowed to find the best alignment [24]. A large window can increase processing time of the search and allow invalid matching between distant points. A small window, by contrast, could miss the best solution. Figure 6 demonstrates that with Euclidean distance, the dips and peaks in the two time series are misaligned and not matched, whereas DTW detects their alignment with their corresponding points from the other time series. While DTW is a more robust distance measure than Euclidean Distance, it is also more computationally intensive. [18] proposed an indexing scheme for DTW that allows faster retrieval. Nevertheless, DTW is still at least several orders slower.

In this approach, signal instances in the test data are presented for an entire activity for classification. An activity is one of the 25 actions (grips or movements) listed in Table 2 and consists of a sequence of consecutive words from one single repetition. For example, a group of the action ‘hammer raise’, HR, consists of a sequence *hr*_1_,*hr*_2_,…*hr*_*m*_,*m*≤40, collected in that order during one of the 12 hammer raise repetitions. Activities in the dataset were truncated to a maximum of 40 words since that is the maximum number of words in the movements and the significant parts of the grips occur in the first 40. The test action is then compared with all actions in the training data starting with only the first word in the test sequence (*hr*_1_), then the first and second (*hr*_1_,*hr*_2_), then the first through third (*hr*_1_,*hr*_2_,*hr*_3_), and so on until all words in that test action are compared (*hr*_1_…*hr*_*m*_). The size of the comparison is limited to either *m*, the number of words in the test sequence, or the number in the activity sequence from the training dataset if shorter.

For each comparison, the DTW is measured using a modified version of [25] and a *K*-nearest-neighbor classification (*K*-nn) scheme used to determine the class. Here *k*=1 was computed and recorded for each comparison window ranging from 1…*m*. A warping window of ±5 was specified, for a total width of 11. The distance measure is a modified lexical distance between the test and training actions. The individual test and training words are compared one letter at a time and their differences are summed as follows:

4 $$  D(a,b) = \!\sum_{i=1}^{8} d\left(a_{i},b_{i}\right), \,\,d(a_{i},b_{i})= \left\{ \begin{array}{l} {0, a_{i} = b_{i}}\\ {|a_{i} - b_{i}| - 1, a_{i} \neq b_{i}}\\ \end{array} \right.   $$

For example,

$$\begin{aligned} &D(`AAAAAAAA',`AACCBBEE')\\ &\quad= 0+0+1+1+0+0+3+3 = 8. \end{aligned} $$

Computing the modified lexical distance in this way avoids the problem of assigning a distance of one to two adjacent values where both are close to the boundary between them and whose value would be much closer to zero than one [24].

This approach matches signal sequences whose shapes are similar but slightly out of line. The window parameter controls the flexibility of the match and the trade-off of large-versus-small was previously discussed. The approach also measures how soon a test sequence can be correctly recognized since the comparison is done in increasing numbers of words in a particular action. The results and discussion sections discuss this.

## Results

Table 4 shows the overall accuracy for all subjects for the first Learning Approach using various classifiers from Approach 1. The Weka workbench was used to build the classification model and evaluate using stratified ten fold cross-validation. The parameters for the classifiers that require them were set to values yielding the highest accuracy using the previously described grid search method. Table 4 shows accuracy for separate runs performed for each subject and the average for the three.

**Table 4:** Overall accuracies for the classifiers in the first learning approach for each subject and average of the three

Method	Subj1 (%)	Subj2 (%)	Subj3 (%)	Avg. (%)
Dec.Tree (C4.5)	76.77	65.18	65.28	69.08
1-NN	77.02	71.51	64.41	71.00
RandFor. (25)	84.28	72.71	72.93	76.64
SVM	80.58	71.19	70.21	74.00

**Fig. 7:**
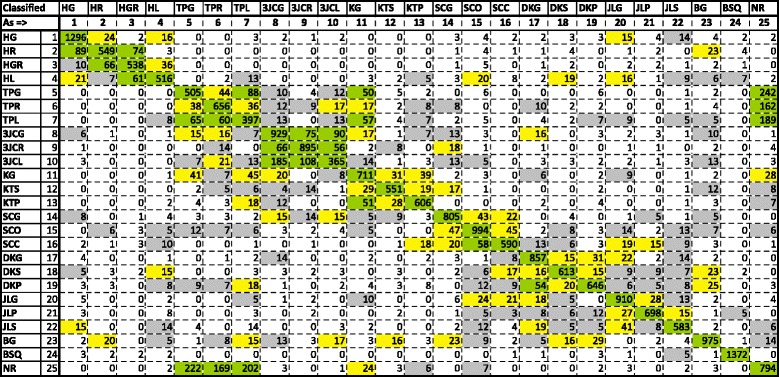
Combined confusion matrix for all 3 subjects for Approach 1 - Random Forest. Classes listed in the rows are the ground truth for one word instance. The total number of predicted classes for all subjects are shown in the cells corresponding to the class in the columns. Higher numbers (50+) are shown in *green*, moderate numbers (15–49) in *yellow*, smaller (5–14) in *gray*, and fewer than 5 in *white*. Note strong *green* along the diagonal, indicating correct predictions. Many incorrect predictions are clustered near their action group (e.g., hammer, tip pinch, 3-jaw-chuck). Note the confusion between the tip pinch group (TPx) and neutral/rest (NR)

**Fig. 8:**
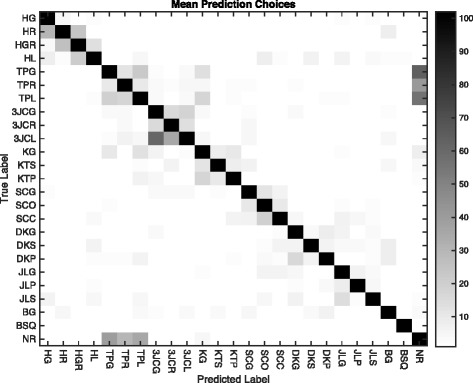
*Black and white* colorgraph rendering of the Random Forest Confusion Matrix from Fig. 7. *Darker* cells indicate higher numbers of correct predictions, which can be seen along the diagonal. Most cells are white, indicating zero predictions. Note that most predictions are close to the diagonal, and many incorrect classifications are clustered around common activity groups (tip pinch, 3-jaw chuck, etc.). A notable exception is the confusion between the tip pinch group (TPx) and neutral/rest (NR)

Random Forest with 25 trees (RF25) had the highest, with an average of 76.64 % for all 25 classes. RF25 was the highest for each of the individual subjects as well. Figure 7 is a confusion matrix showing combined subject results. The individual confusion matrices for each of the subject’s RF25 run were added and the resulting matrix used as the basis for the figure. Figure 8 is a gray scale colorgraph version of the confusion matrix.

For the second Learning Approach, Affinity Matrix, a custom MATLAB^®;^ solution was developed. Unlike Approach 1, which only classified individual words, this one sums the computed affinity value for the instance being classified with the values of the previous *w* values from the input stream.

To evaluate the approach, the eight action group datasets are divided into 12 segments, one for each action repetition. Segment one, for example, includes the words in the first repetition of the hammer action group (HG, HR, HGR, and HL), followed by the tip pinch group (TPG, TPR, and TPL), then 3-jaw chuck (3JCG, 3JCR, and 3JCL), etc. Likewise, the second segment includes the second repetition of all action groups. Neutral/rest (NR) words from seconds 1–3 for each of the action group runs was added to each of the 12 runs. This was done to inject a representative sample of that posture (≈6 *%* of total) since including all NRs would result in their being 40 % of the total and unbalancing the dataset. Twelve separate classification training and testing runs were performed. Each segment was withheld as a test set for a run one time, with the remaining 11 used to build the Affinity Matrix. Accuracies from classifying the test sets in the twelve runs were averaged.

**Fig. 9:**
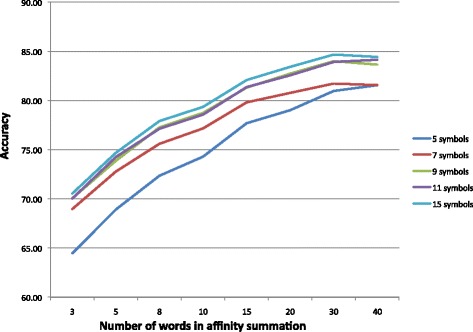
Graph of the Affinity Matrix approach for the average accuracy of all subjects for various numbers of symbols and words used in the affinity summation. The accuracy for 9, 11, and 15 symbols is tightly clustered and therefore similar, but superior to trials using only 5 and 7. Improvement flattens out in the vicinity of 20 to 30 words for all numbers of symbols

**Fig. 10:**
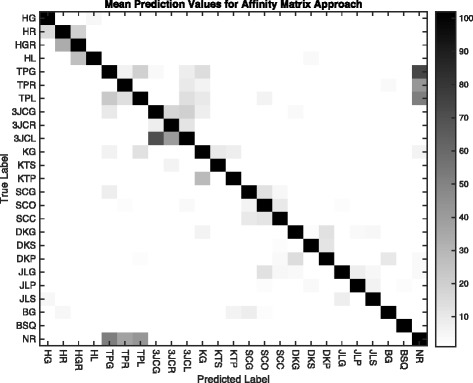
Confusion matrix showing an overlay for all three subjects for the Affinity approach using data from the best Approach 2 test run for each subject. Darker cells indicate higher numbers of correct predictions, which can be seen along the diagonal. Most cells are *white*, indicating zero predictions. Note that most predictions are close to the diagonal, and many incorrect classifications are clustered around common activity groups (tip pinch, 3-jaw chuck, etc.). A notable exception is the confusion between the tip pinch group (TPx) and neutral/rest (NR)

This approach requires two parameters: the number of SAX symbols used to discretize the signals, and *w*, the number of words immediately before the test instance used to create the affinity sum. The number of symbols was varied from 5 to 15, and the number of words from 3 to 40. Table 5 shows the various parameter combinations. The table contains the high-low range for the three subjects for each combination. The graph in Fig. 9 shows the average accuracy for the three subjects for word counts up to 40. The lines for symbols 9, 11, and 15 are tightly clustered and superior to 5 and 7 for all word counts. Improvement flattens out between 20 and 30 words, indicating parameter settings that will produce maximum accuracy. Figure 10 is a confusion matrix showing combined 3-subject results for the Affinity Matrix approach. As with the first approach, the individual confusion matrices for each of the subjects were added and the resulting matrix used as the basis for the figure. Each subject’s best Approach 2 run was used in the summation, generally for 11 SAX symbols and 30 words.

**Table 5:** Affinity result ranges among the 3 subjects for selected numbers of SAX symbols and number of words *w* used in affinity summation

# symbols	3	5	10	15	30
5	[61.0, 70.6]	[65.7, 74.6]	[71.7, 79.4]	[75.0, 82.6]	[77.6, 85.6]
7	[65.4, 75.9]	[69.5, 79.8]	[73.8, 83.0]	[76.5, 85.5]	[78.0, 86.8]
9	[67.1, 75.5]	[70.9, 79.4]	[75.7, 83.5]	[78.2, 85.6]	[80.7, 88.5]
11	[66.6, 76.2]	[71.4, 79.7]	[75.8, 83.8]	[78.2, 86.0]	[81.0, 88.4]
15	[67.1, 77.2]	[71.4, 80.7]	[75.9, 85.5]	[78.9, 87.8]	[81.4, 90.0]

For the third Learning Approach, Dynamic Time Warping (DTW), the data was segmented as described for the second approach. Unlike that approach, which classified individual words based on summed affinity values of immediately preceding words, this approach only classifies activity segments, as described in the DTW approach section. This approach only requires one parameter: number of SAX symbols. However, since the comparison is made for an increasing number of words, the success rate at each word count is noted and reported. Results are therefore shown for various combinations of SAX symbols and word counts.

**Fig. 11:**
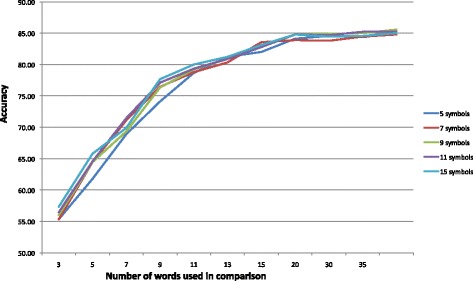
Graph of the Dynamic Time Warping approach for the average accuracy of all subjects for various numbers of symbols and words participating in the comparison. The accuracy flattens out in the vicinity of 20 words for all numbers of symbols. The number of SAX symbols used doesn’t have much affect in the accuracy

**Fig. 12:**
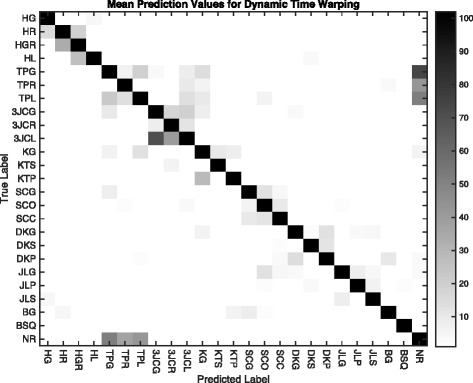
Confusion matrix showing an overlay for all three subjects for the DTW approach using data from the best test run for each subject. As with the Affinity approach, most predictions are close to the diagonal (*dark* cells), and many incorrect classifications are clustered around common activity groups (tip pinch, 3-jaw chuck, etc.). Similar to the Affinity approach is the confusion between the tip pinch group (TPx) and neutral/rest (NR)

Table 6 shows results for the various parameter and word count combinations. The table contains the high-low range for the three subjects for each combination. The graph in Fig. 11 shows the average values for the three subjects for word counts up to 35. The lines for all symbol values are tightly clustered and improvement flattens out at 20 words. Figure 12 is a confusion matrix showing combined 3-subject results for the DTW approach. As with the other approaches, the individual confusion matrices for each of the subjects were added and the resulting matrix used as the basis for the figure. Each subject’s best Approach 3 run using 15 symbols and measured at 20 words was used in the summation.

**Table 6:** Dynamic Time Warping result ranges among the 3 subjects for selected numbers of SAX symbols and number of words used in the comparison

# symbols	3	7	11	15	20	
5	[53.7, 57.0]	[64.0, 72.0]	[74.7, 81.0]	[78.3, 85.7]	[83.0, 85.7]	
7	[52.7, 59.3]	[64.3, 77.0]	[74.0, 82.7]	[78.3, 88.7]	[80.3, 87.0]	
9	[53.7, 59.0]	[62.7, 77.3]	[74.3, 84.7]	[78.0, 88.3]	[81.3, 88.7]	
11	[55.3, 57.3]	[65.3, 78.3]	[76.7, 83.3]	[80.0, 86.7]	[82.7, 89.0]	
15	[56.3, 58.3]	[64.7, 75.3]	[76.3, 84.0]	[79.3, 87.7]	[81.7, 88.7]	

Table 7 lists comparative accuracies for all approaches. The first four rows list accuracies for each subject for the classification methods used in the first approach. The last two rows are values for the second and third. In the latter two cases, values for each subject’s best runs were selected. The Affinity Approach is superior to all Approach 1 classifiers for each subject and the average, and in the case of subjects 2 and 3, significantly better. The DTW Approach was superior to the Affinity Approach for all except subject 1, which showed equivalent accuracy, and significantly superior to the Approach 1 classifiers across the board.

**Table 7:** Overall accuracies for the classifiers in all learning approaches

Method	Subj1 (%)	Subj2 (%)	Subj3 (%)	Avg. (%)
Dec.Tree (C4.5)	76.77	65.18	65.28	69.08
1-NN	77.02	71.51	64.41	71.00
RandFor. (25)	84.28	72.71	72.93	76.64
SVM	80.58	71.19	70.21	74.00
Affinity	88.91	81.95	79.94	83.63
DTW	88.67	83.67	82.67	85.22

The first four are the classifiers used in the first approach, while the bottom two rows represent values for the second and third approaches at the top of the curve for each subject, as noted in Figs. 9 and 11

## Discussion and conclusions

To represent the grips and movements in Table 2 - prehensile patterns - effectively, we accepted the challenge of having to select both electrical signal data from multiple extrinsic muscles of the hand as well as position data obtainable from the accelerometer. We recognize that not all relevant signals contributing to prehensile activity were captured from a few surface electrodes - deeper muscles may not be adequately represented. We selected an appropriate set of functional prehensile patterns likely to be useful in informing clinicians about which muscles and hand/wrist movements associate with these activities. We further believe that with further improvements they can form the basis for applications involving myoelectric control of hand grips and movements.

The choice of which methods to use to reduce the captured data and recognize the patterns was empirical. We approached it from the perspective of trying to identify the “best fit”. We elected to use an MAV window of 100 ms as the basic atomic learning instance and standard classifiers (Decision Tree, Nearest-neighbor, Support Vector Machine, and Random Forest). In this first approach, the classifiers were trained to recognize individual 100 ms instances without considering any other information. The best performer, Random Forest (25 trees), yielded an average accuracy of 76.64 % for all 25 classes. While this is comparable to results obtained elsewhere for similar problems, it is less than what would be needed to be useful in real life scenarios. Besides having a too-low accuracy, a difficulty arises when an activity consisting of multiple 100 ms signal instances results in an inconsistent stream of predicted movements. How would a stream of {HR, HR, HL, HR, JLS, HR, JLS } be interpreted? Smoothing the result stream, as reported in [16] may help, but won’t erase all ambiguity.

The second approach reported in this paper attempted to improve classification accuracy in two ways. The first used the SAX concept to discretize the real-valued signals into a set of symbols. This had the effect of normalizing the data while reducing the total signal space to a finite number of symbol combinations. It also allows for an easier visual interpretation of signal values. For example, it’s plain to see that for a five symbol alphabet ’EEEEEEEE’ represents a set of high signal values compared with ‘AAAAAAAA’.

The second improvement injected time-context into the process of classifying a signal by considering immediately preceding values in the stream. Affinity values for a single 100 ms instance can reflect a strong probability for a specific class, in some cases with a 90 % + probability for one class and low or zero values for the rest. In other cases, the affinity can be spread across ten or more classes with a strong preference for none and the selected class’s affinity being below 20 %. In those cases, the case for selecting the class with the highest affinity is weak. We attempted to improve the decision by calculating the sum of the class values for the current with some number of previous instances to better reflect the “sense of the neighorhood” in terms of identifying the true class. Experimenting with various combinations of SAX symbols and numbers of words in the affinity summation led to an improved average accuracy of 83.63 % for 11 symbols and 30 words.

While the Affinity approach improves prediction accuracy using information from adjacent 100 ms instances, the third approach considered the entire movement. This approach also relied on converting the signal values to SAX symbols prior to classifying. The approach segmented the data stream into the 25 activities listed in Table 2. It also employed the Dynamic Time Warping concept to account for small time shifts in the signals and allow for a relaxed and more realistic comparison. In this approach we experimented with various numbers of SAX symbols and noted the accuracy for each numbers of words used in the classification. Surprisingly, the number of SAX symbols had little impact on accuracy, but increasing the number of words up to 20 improved accuracy before levelling off. At that level the average accuracy was 85.22 %.

## Conclusions

In summary, Approach 2 (Affinity) improved on the classifiers used in Approach 1, and Approach 3 (DTW) improved on 2. Including context information from the signal stream helps, and considering entire movement sequences helps even more. This isn’t surprising since the goal is to recognize complete movements, and not small slivers of movements. Incorporating a wider swath of data improves this recognition.

How practical would the second and third approaches be in real-life situations? For real-time control systems Approach 1 only requires collection and analysis of a 100 ms signal slice before rendering a classification decision. The timing requirement would be met, at the cost of lower overall accuracy and some inconsistency in the predicted signal stream classes. Approach 2 only requires consideration of the current signal instance and some that were already seen, and yields higher accuracy while meeting timing requirements for real-time applications. Approach 3 yields the highest accuracy, but requires that an entire activity segment be considered. However, the results show that not all signal words in the entire activity segment need to be collected and included in the classification. In fact 20 words, or one second of signal data, suffices to reach maximum accuracy. A one second delay is likely too long in most real-time applications and would have to be shortened to consider this approach in those settings. For applications without a real-time requirement, the thirs approach is usable and may be considered.

The findings reported here support the view that prehensile patterns can be distinguished by combining electrical and mechanical properties of the task. This is both clinically useful and opens the way for an approach to help simulate hand functional activities. With improvements it may also prove useful in real time control applications.

Future work should address some of the shortcomings of the approaches reported here. More prehensile patterns should be investigated, leading to the goal of recognizing continuous movement, not just discrete action segments. A step toward achieving that goal is to create a more wholistic model that combines the electric signal, the mechanical components, and the dynamic components to “picture” the activity in its entirety.

Exploring the recognition of individual tasks and their differences in accuracy would be useful in breaking down the total prehensile space into those that can be easily recognized and those that can’t. In this research the ball squeeze (BSQ) and key turn-supination (KTS) were well-recognized with high true positive rates for all methods. Tip pinch grip (TPG) and key grip (KG), by contrast proved difficult to recognize. For the difficult cases, additional analytical tools can be considered such as recognizing family of movements organized around their base grip. For those, a hierarchical strategy could be used to recognize the family, for example a hammer grip, and then operate only on those instances belonging to that family in a secondary step to identify the specific movement involved such as hammer raise and lower.

Finally, this paper presented research based on data collected from a three able-bodied subjects. Future work should involve more subjects, including those with upper extremity disability. Including less than fully-abled subjects could lead to discovering myoelectric differences that could aid diagnoses and suggest treatment options.
